# Differential expression profiles and functional prediction of circRNA in mice with traumatic heterotopic ossification

**DOI:** 10.3389/fimmu.2022.1090529

**Published:** 2023-01-12

**Authors:** Zheng Wang, Xinzeyu Yi, Yuhang Liu, Qiaoyun Liu, Zonghuan Li, Aixi Yu

**Affiliations:** Department of Orthopedics Trauma and Microsurgery, Zhongnan Hospital of Wuhan University, Wuhan, China

**Keywords:** Achilles tenotomy, heterotopic ossification, circRNAs, microarray, miRNAs, circRNA-miRNA-mRNA interaction network

## Abstract

**Background:**

Traumatic heterotopic ossification (HO) is an intractable sequela incited by inflammatory insult. To date, the exact molecular mechanisms of traumatic HO formation remain unclear. Recent studies have indicated that circular RNAs (circRNAs) participate in various human skeletal diseases. Although the formation of HO recapitulates many programs during bone development and remodeling, few data are available concerning whether circRNAs could participate in this pathological osteogenesis.

**Methods:**

To investigate the differentially expressed circRNAs (DE-circRNAs) in HO formation, microarray assay was performed to analyze the circRNA expression profile in four pairs of mice HO tissues and normal tissues. Then, qRT-PCR was applied to verify the microarray data. Gene Ontology (GO) and Kyoto Encyclopedia of Genes and Genomes (KEGG) analyses showed the biological functions of the differentially expressed circRNAs target genes. Cytoscape software was used to construct the circRNA–miRNA–mRNA network for circRNAs with different expression levels as well as the target genes.

**Results:**

We demonstrated that 491 circRNAs were significantly differentially expressed in mouse HO tissues by a fold-change ≥ 2 and p-value ≤ 0.05. Among them, the expressions of 168 circRNAs were increased, while 323 were decreased. The expression levels of 10 selected circRNAs were verified successfully by qRT-PCR. GO analysis exhibited that these DE-circRNAs participated in a series of cellular processes. KEGG pathway analysis revealed that multiple upregulated and downregulated pathways were closely related to the DE-circRNAs in HO mice. The circRNA-miRNA-mRNA networks demonstrated that DE-circRNAs may be involved in the pathological osteogenesis of HO through the circRNA-targeted miRNA-mRNA axis.

**Conclusion:**

Our study first demonstrated the expression profiles and predicted the potential functions of DE-circRNAs in mice traumatic HO, which may shed new light on the elucidation of mechanisms as well as provide novel potential peripheral biological diagnostic markers and therapeutic targets for traumatic HO.

## 1 Introduction

Traumatic heterotopic ossification (HO) is defined as the pathological formation of bone and cartilage matrix in soft tissues, which could occur in the context of arthroplasty, bone fractures or dislocations, traumatic brain and spinal cord injuries, and severe burns (1). This intractable disorder can lead to severe symptoms and consequences, including chronic pain, reduced range of joint motion, impaired prosthetic wearing, neurovascular compression, and skin damage, resulting in poor quality of life (2). Currently, surgical excision of the lesion is the only standard therapeutic option for symptomatic HO, but it exhibits a high recurrence rate (3). Other prophylactic measures are limited to radiation therapy and nonsteroidal anti-inflammatory drugs, such as indomethacin, which can result in severe complications (4). To date, the exact molecular mechanisms of traumatic HO formation remain unclear, leading to the stagnation in the development of safe and effective treatments.

Histologically, traumatic HO is believed to develop through a process of endochondral ossification involving four stages: inflammation, chondrogenesis, osteogenesis, and maturation stages (1, 5, 6). Recently studies have demonstrated that Inflammatory cells, specifically myeloid cells, play a major role in this form of pathological osteogenesis (7, 8). Enrichment of macrophages has been identified in HO tissues and both M1 and M2 macrophages are involved in facilitate HO formation in different ways (8). After acute inflammatory insult, tissue-resident mesenchymal progenitor cells (TMPCs) are recruited and located in the injury site (9, 10). Simultaneously, local macrophages produce TGF-β1 that has been identified to accelerate chondrogenesis and osteogenesis in TMPCs, which is a fundamental process for endochondral ossification (7, 8). In addition, many studies have revealed that macrophages and their secreted cytokines are important regulators in fracture healing and bone development (11, 12). Therefore, these observations during the process of traumatic HO formation recapitulate many programs of bone development and remodeling (4), suggesting overlapping mechanisms between developing bone and HO, such as the intimate coupling of circular RNAs (circRNAs) and osteogenesis (13–15).

CircRNAs are a group of non-coding RNAs with a covalently closed continuous loop which are highly conserved and commonly found in mammalian cells (16). Most circRNAs in eukaryotic cells are spliced from exons and located in the cytoplasm and their primary regulatory mode is to act as miRNA sponges, which can competitively bind miRNA to relieve the inhibition of miRNA on its downstream target genes, thereby increasing the expression level of target genes (17). The roles of circRNAs in the development of skeletal diseases, including bone defects, osteoporosis, osteoarthritis and osteonecrosis of the femoral head (ONFH), have been reported (18, 19). During the initiation and development of these diseases, the circRNA-miRNA-mRNA regulatory axis plays a major role in regulating the osteogenic differentiation of stem cells from different origins. For instance, Yu et al. indicated that the knockdown of circ_0003204 could promote the osteogenic differentiation of human adipose-derived stem cells through regulating miR-370-3p/HDAC4 axis in repairing bone defects (20). Li et al. found that circRNA_0001795 could sponge miRNA-339-5p to increase the expression of yes-associated protein 1 and facilitate the osteogenic differentiation of bone marrow mesenchymal stem cells (BMSCs) in attenuating osteoporosis progression (21). Feng et al. demonstrated that circHGF could inhibit the binding of miR-25-3p to SMAD7, thus suppressing the osteogenic differentiation of BMSCs and leading to ONFH (22).

**Figure 1:**
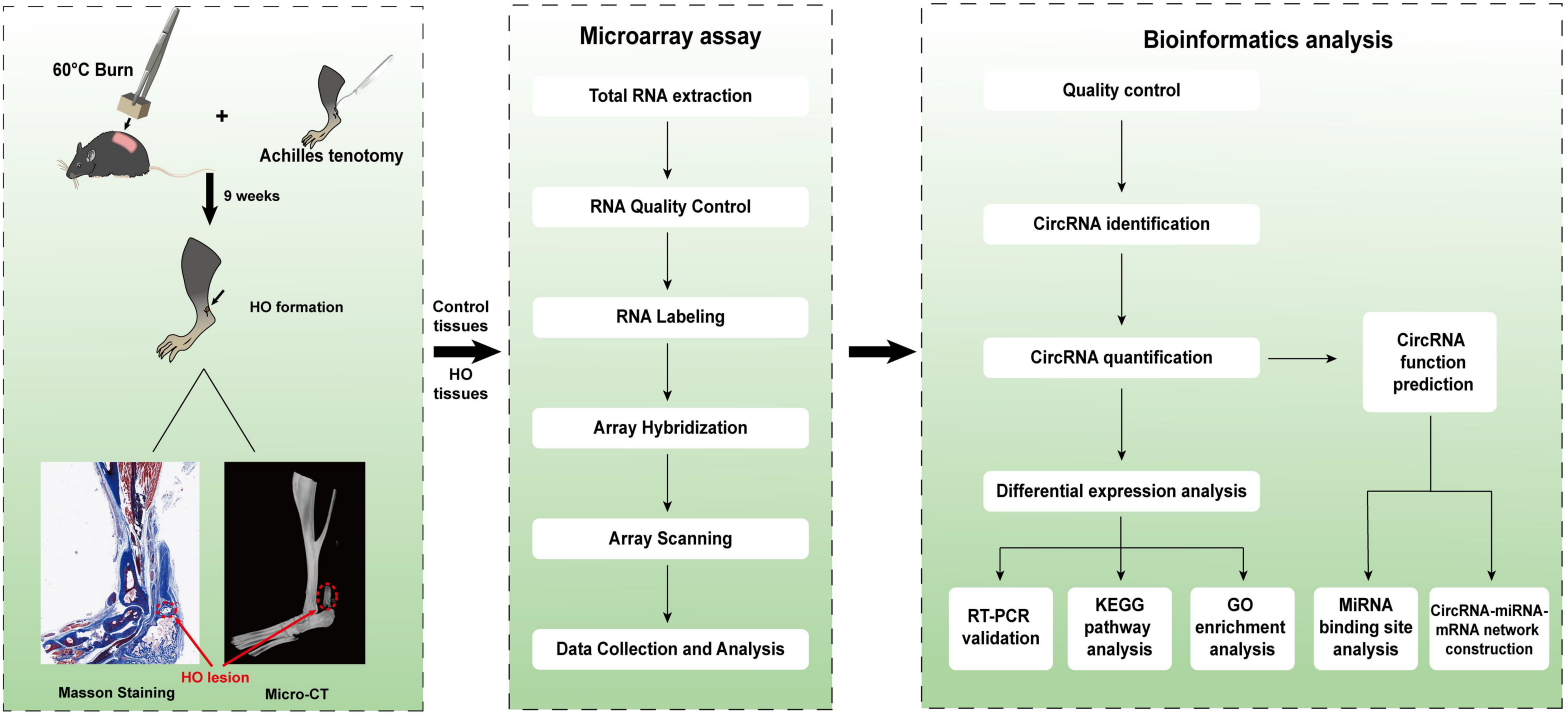
Schematic diagram of the analytical procedures used in this study.

However, few data are available concerning whether circRNAs could participate in the pathological osteogenesis of HO. In this study, we established a mouse traumatic HO model, and then performed the microarray assay to examine the expression profiles of circRNAs in the ectopic bone lesions from HO mice. In addition, we predicted the potential functions of the differentially expressed circRNAs (DE-circRNAs) by Gene ontology (GO) and Kyoto Encyclopedia of Genes and Genomes (KEGG) pathway analyses as well as constructing the circRNA-miRNA-mRNA networks. The procedures of the whole experiment are shown in **Figure 1**.

## 2 Materials and methods

### 2.1 Animals

Eighteen male Wild-type C57BL/6 mice (aged 6-8 weeks, weighing 18–20 g) were purchased from the Sibeifu Biotechnology Co., Ltd. (Beijing, China). Animals were maintained in a temperature-regulated (23-25°C) and humidity-controlled (50% relative humidity) room with a light/dark cycle of 12 h day/night. Mice were allowed free access to food and water. All animal experiments were conducted according to the guidelines for the Care and Use of Laboratory Animals of the National Institutes of Health, and were subjected to the approval by the Experimental Animal Welfare Ethics Committee of Zhongnan Hospital of Wuhan University.

### 2.2 Establishment of mice traumatic HO model

**Figure 2:**
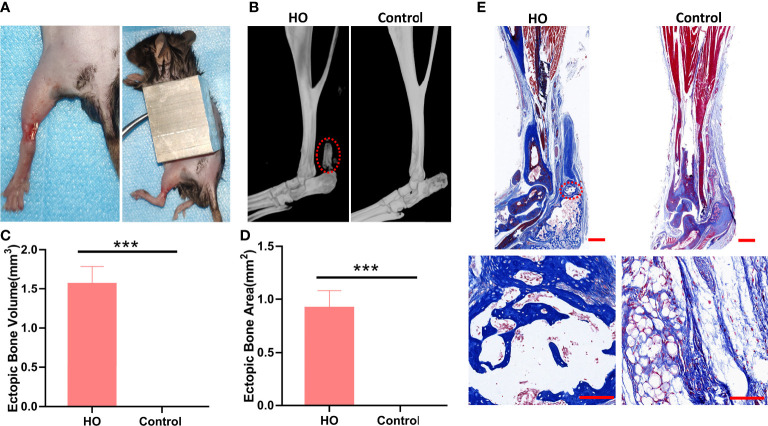
Identification of the HO model. **(A)** Surgical procedures for induced traumatic HO by Achilles tenotomy and burn injury. **(B)** Micro-CT reconstruction images of Achilles tendon (red circle) in HO and control groups 9 weeks after surgery. **(C)** Micro-CT quantifications of ectopic bone volume in the injury sites (n = 4). **(D)** Masson staining of Achilles tendon in each group 9 weeks after surgery. The ectopic bone appears dark blue (red circle), while the tendon appears light blue. Original magnification: ×1 (top row); ×20 (bottom row). **(E)** Histomorphometric quantifications of ectopic bone areas in the injury sites (n = 4). *** indicates that p < 0.001.

The mice were randomly divided into a HO and a control group (n = 9). The mice in the HO group underwent Achilles tenotomy and skin burn to induce ectopic bone formation at Achilles tendon sites (**Figure 2A**), as previously described (23). After being anesthetized by intraperitoneal injection of pentobarbital sodium (5 mL/kg; Sigma-Aldrich, USA), a longitudinal incision was performed along the medial aspect of the left Achilles tendon. Then, shaving the dorsum of the mouse and exposing the skin. Mice received a 30% total body surface area partial-thickness burn on the shaved dorsum, with an aluminum block weighing 35 g with approximate measurements 2 cm x 2 cm x 3 cm heated to 60°C in a water bath for 17 sec. In order to achieve the consistency of burn depth in mice, ensure that the entire surface area of the block is in contact with the mouse and avoid additional pressure other than gravity to the block. The mice in control were only subjected to a longitudinal skin incision without skin burn and Achilles tenotomy. The left leg incision was closed in layers using 4-0 sutures in both groups. Animals were injected subcutaneously with Meloxicam analgesic (0.2 mg/kg) after surgery.

### 2.3 Micro-CT and histological analysis for HO formation

At 9 weeks after surgery, the left hind limbs were collected from both groups for scanning using a high-resolution micro-CT system (SkyScan 1176; Bruker microCT, Kontich, Belgium) to examine the HO formation (n = 5). Scans were conducted with the same settings: 89-kV polychromatic x-ray beam, 256 μA current and an exposure time of 81 milliseconds per 180o rotation. Images were processed using NRecon Reconstruction software (Bruker) to align scan images and generate reconstructed 3D and cross-sectional images. Total new bone (differential new bone from native bone) formation was calculated using Bruker micro-CT volumetric software (version 1.14.10.0). After micro-CT analysis, the left hind limbs were processed and stained with Masson’s Trichrome Stain Kit (Baiqiandu, Wuhan, China) following the manufacturer’s instructions. The sections were visualized under a light microscope (Olympus, Japan). Images were captured using an optical Lightools Imaging System and the ectopic bone areas were calculated using the ImageJ software 1.8 (Bethesda, MD, USA).

### 2.4 RNA labeling and assay hybridization

At 9 weeks after surgery, tissues were harvested from the tendon insertion into the calcaneus to the distal gastrocnemius in both groups (n = 4). The TRIzol reagent (Invitrogen Life Technologies, CA, USA) was applied to extract the total RNA from HO lesions and normal tissues. Sample labelling and array hybridization were conducted according to the manufacturer’s protocol (Arraystar, MD, USA). Firstly, in order to remove linear RNAs and enrich circular RNAs, Rnase R (Epicentre, WI, USA) was used to digest the total RNAs. Then, the enriched circular RNAs were amplified and transcribed into fluorescent cRNA *via* a random priming procedure with Arraystar Super RNA Labeling Kit (Arraystar). Thereafter, purifying the labeled cRNAs (pmol Cy3/μg cRNA) with the RNeasy Mini Kit (Qiagen, Dusseldorf, Germany) and then measuring the concentration and specific activity of the labeled cRNAs by the NanoDrop ND-1000. Subsequently, 5 μl 10 × blocking solution and 1 μl of 25× fragmentation buffer was applied to fragment each labeled cRNA (1μg) at 60°C for 30 min. After that, 25 μl 2× hybridization buffer was added to dilute the labeled cRNA. Then, 50 μl of hybridization solution was allocated into the gasket slide and assembled to the circRNA expression microarray slide. The slides were heated at 65°C for 17 hours in an Agilent Hybridization Oven. Finally, the hybridized arrays were washed, fixed and scanned with the Agilent Scanner G2505C (Agilent Technologies, CA, USA).

### 2.5 Microarray analysis of circRNAs

Scanned images were imported into Agilent Feature Extraction software for raw data extraction. With the help of the R software limma package, we performed a differential analysis of these circRNAs. After quantile normalization of the raw data, we conducted the low-intensity filtering and then selected the circRNAs with flags in Present or Marginal (“All Targets Value”) for further analyses. Fold change filtering was applied to identify differentially expressed circRNA (DE-circRNAs) between HO lesions and normal tissues. Student’s t-test was employed to evaluate the statistical significance of the difference. CircRNAs with fold changes ≥2 and *P* ≤ 0.05 are selected as the significantly differentially expressed.

### 2.6 Quantitative real-time PCR validation

Ten DE-circRNAs including five upregulated and five downregulated circRNAs were randomly selected to verify the authenticity of the microarray data, as shown in **Table 1**. Quantitative real-time PCR (qRT-PCR) was conducted using a 2× PCR master mix (Arraystar) on a QuantStudio5 Real-time PCR System (Applied Science, USA). The specific primer pairs used in the study are listed in **Table 1**. The reaction program was set as follows: for 10 min at 95°C, and 40 PCR cycles (95°C, 10 s and 60°C, 60 s (fluorescence collection)). U6 was used as a housekeeping gene for standardization. The relative expressions of circRNAs were calculated with the 2-ΔΔCt method.

**Table 1:** The primer list was used for real-time quantitative PCR.

Gene name	Bidirectional primer sequences (5’-3’)	The length of the product (bp)
U6	F:GCTTCGGCAGCACATATACTAAAAT R:CGCTTCACGAATTTGCGTGTCAT	89
mmu_circRNA_43813	F:AGGTCAGAAGATAGTTTTCCCAGG R:TTCTGCTGCTATTTTTCGTTCC	73
mmu_circRNA_34414	F:AGAACCCGAGGGAGACCTTATC R:CTAAGAAGATGGTGAACCTCTGAGC	179
mmu_circRNA_21813	F:AATACTGGTTTGCTGTTCCCC R:ACTATCTCTGCATTTTGTTTGGC	191
mmu_circRNA_24245	F:TTACTTTGCCACATTGGAGTATA R:TCTGTTAGGGATTTTAGGTGGA	172
mmu_circRNA_37783	F:CCACCACCACGACGAAGACT R:CTGCCTACCTCACCGTTCTTTAC	90
mmu_circRNA_36033	F:CGTCCACGAGTATGTGCTTTA R:CATCAGGGAGGCATCCAGGT	139
mmu_circRNA_000595	F:TCAGCGATTTATACAACGAGGCT R:CACTGTCGCCACTGGATTCA	77
mmu_circRNA_35735	F:TGTTTCCCGCATCTACGCA R:ACGAAGGAGGCATCCGACTG	203
mmu_circRNA_41256	F:GGACATCGTAGGCTCTGAGG R:TCCGTAGGTAGTTTTGGAAATC	100
mmu_circRNA_41257	F:AGGAGGGCGGGGAAAATG R:GTAGTCTTCGTCCAGTTTGCTCAA	81

### 2.7 Statistical analysis

Statistical analyses were performed using GraphPad Prism 7.0 (GraphPad Software, CA, USA). All relevant data are expressed as the mean ± standard deviation (means ± SD). Student’s t-test was applied to evaluate the difference between the two groups. Statistical significance was set at *P* < 0.05.

### 2.8 Bioinformatics analysis

To predict the function of the target genes of circRNAs, GO and KEGG pathway analyses were performed for further bioinformatics analysis. The circRNA/miRNA interaction was predicted with Arraystar’s home-made miRNA target prediction software based on TargetScan and miRanda. CircRNA-miRNA-mRNA regulatory networks were established by Cytoscape.

## 3 Results

### 3.1 Validation of the establishment of traumatic HO model

At 9 weeks after surgery, we evaluated the pathological formation of bone matrix in the Achilles tendon. Micro-CT examinations indicated that all mice underwent both Achilles tenotomy and skin burn injury developed traumatic HO, with obvious circular high-density shadow at the surgical site, and the average volume of ectopic bone was (1.56 ± 0.224) mm3 (**Figures 2B, C**). In contrast, no mice developed traumatic HO in the control group. Masson staining showed the ectopic bone formation in the HO group with bone trabecular and bone marrow structures (**Figures 2D, E**), which further proved that the mice HO model was successfully established.

### 3.2 Expression profiles of altered circRNAs in HO tissues

**Figure 3:**
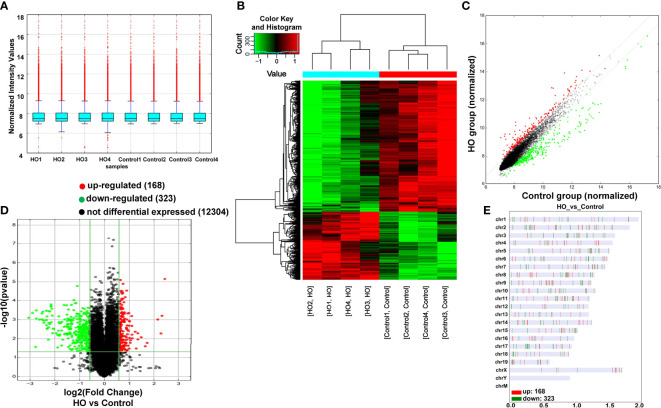
Differentially expressed profile of circRNAs between HO and control samples. **(A)** The box plot shows the distribution of circRNAs between HO and control samples. **(B)** The Hierarchical clustering plot indicates the differentially expressed circRNA profiles in the eight samples. “Red” represents the higher expression, while “green” represents the lower expression level. **(C)** The scatter plot demonstrates the differentially expressed circRNA profiles in the eight samples. **(D)** The volcano plot exhibits the distinguishable circRNAs expressions. **(E)** Chromosomal distributions of circRNAs in HO and control samples.

Four pairs of mice HO tissues and normal tissues were analyzed using microarray assay to characterize the expression profile of circular RNA in HO tissues. The box plot demonstrated that the distribution of normalized intensity values was similar after normalization in the tested samples (**Figure 3A**). The hierarchical clustering revealed diacritical circRNA profiles from four pairs of samples on the ground of their expression level (**Figures 3B, C**), suggesting that circRNAs have different expression profiles between HO lesions and normal tissues. The volcano plot was established to identify the significant differences between HO and control groups (fold change ≥ 2, *P* < 0.05) (**Figure 3D**). Totally 491 circRNAs were significantly altered in HO lesions compared with normal tissues. Of which, 168 circRNAs were upregulated and 323 circRNAs were downregulated. Among them, mmu_circRNA_29625 showed the highest fold change in expression level, while mmu_circRNA_018683 showed the lowest. The distribution of the DE-circRNAs in chromosomes showed that most of the circRNAs were transcribed from chr2, chr6, chr17, and seldom from chrX, chrY and chrM (**Figure 3E**). The top 10 upregulated and top 10 downregulated circRNAs are shown in **Table 2**.

**Table 2:** Biological information for the top 10 upregulated and downregulated circRNAs.

CircRNA ID	*P*-value	Fold change	Chromosome	CircRNA type	Best transcript	Gene Symbol
Upregulated						
mmu_circRNA_29625	7.08914E-06	5.4093633	chr16	exonic	NM_028295	Pdia5
mmu_circRNA_24245	0.000629008	4.982093	chr11	exonic	NM_145436	Cdc27
mmu_circRNA_45921	0.005279066	4.8450605	chrX	exonic	NM_172783	Phka2
mmu_circRNA_19469	0.000983826	4.4320693	chr7	antisense	NM_009277	Trim21
mmu_circRNA_28874	0.016639406	3.2943937	chr15	exonic	NM_029946	Efcab6
mmu_circRNA_43813	0.024866247	3.1372888	chr9	exonic	NM_146221	Zfp426
mmu_circRNA_007951	0.008395959	2.7962011	chr8	sense overlapping	NM_001081415	Samd1
mmu_circRNA_21813	0.010790482	2.5414307	chr10	exonic	NM_172495	Ncoa7
mmu_circRNA_34414	0.005167793	2.5414159	chr2	exonic	NM_032393	Map1a
mmu_circRNA_37783	0.032643936	2.4375915	chr4	exonic	NM_016799	Srrm1
Down-regulated						
mmu_circRNA_018683	0.00079836	-8.2956948	chr17	sense overlapping	None	–
mmu_circRNA_009370	0.000450913	-7.440702	chr17	sense overlapping	None	–
mmu_circRNA_20066	0.027083247	-6.8369871	chr1	sense overlapping	uc029qoi.1	DQ715306
mmu_circRNA_010749	0.000864345	-6.706647	chr17	sense overlapping	None	–
mmu_circRNA_016863	0.000327654	-6.6996484	chr17	sense overlapping	None	–
mmu_circRNA_005915	0.000992073	-6.1665456	chr17	sense overlapping	None	–
mmu_circRNA_008051	0.000965598	-5.7502166	chr17	sense overlapping	None	–
mmu_circRNA_19450	0.000473563	-5.7329777	chr7	sense overlapping	NM_007590	Calm3
mmu_circRNA_001769	0.001037835	-5.6092089	chr17	sense overlapping	None	–
mmu_circRNA_41204	0.000380114	-5.5755345	chr7	sense overlapping	NM_007590	Calm3

### 3.3 Validation of the microarray data by qRT-PCR

**Figure 4:**
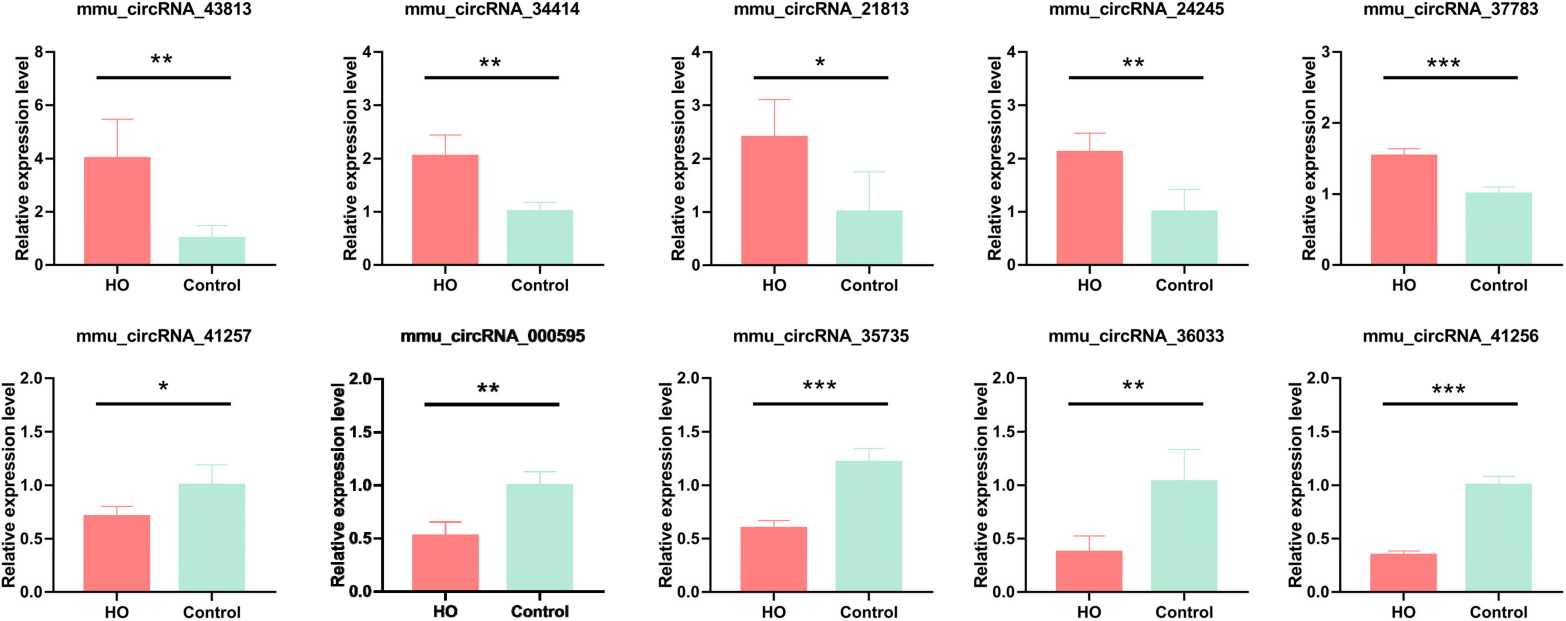
Validation of the randomly selected 10 DE-circRNAs by qRT-PCR. Changes in DE-circRNAs expressions were confirmed using qRT-PCR in the control and HO groups (n = 4). *p < 0.05, **p < 0.01, ***p < 0.001. DE-circRNAs, differentially expressed circRNA.

To further verify the accuracy of the results, 10 DE-circRNAs comprising five upregulated and five downregulated circRNAs were selected at random for qRT-PCR analysis (**Figure 4**). The results revealed that the expressions of mmu_circRNA_43813, mmu_circRNA_34414, mmu_circRNA_21813, mmu_circRNA_24245 and mmu_circRNA_37783 were significantly upregulated in HO tissues, and the expressions of mmu_circRNA_41257, mmu_circRNA_000595, mmu_circRNA_35735, mmu_circRNA_36033 and mmu_circRNA_41256 were significantly downregulated in HO tissues. Thus, the qRT-PCR results were in accordance with microarray analysis, which confirms the reliability of our circRNAs expression profile.

### 3.4 GO and KEGG analyses of the parental genes of circRNAs in HO tissues

**Figure 5:**
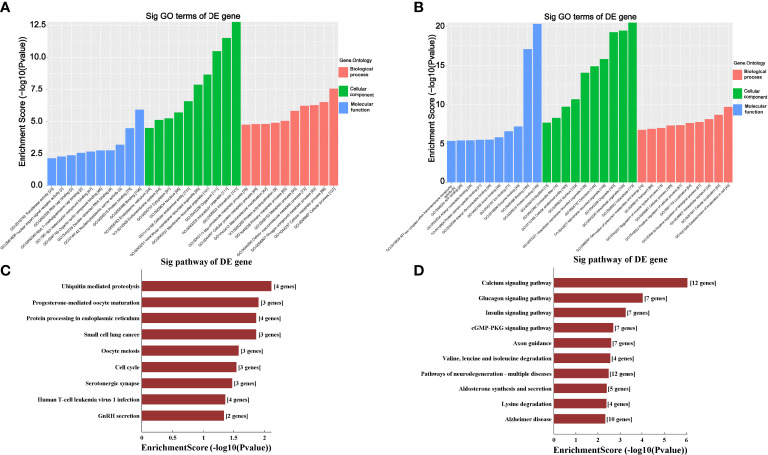
Gene Ontology (GO) and Kyoto Encyclopedia of Genes and Genomes (KEGG) analyses for upregulated and downregulated circRNAs. **(A, B)** GO analysis predicted the top 10 upregulated **(A)** and the top 10 downregulated **(B)** enriched genes in biological processes, cellular components or molecular functions based on their fold enrichment scores. **(C, D)** KEGG pathway analysis predicted the top 10 upregulated **(C)** and the top 10 downregulated **(D)** enriched pathways of DE-circRNAs parental genes. Sig, significantly; DE-circRNAs, differentially expressed circRNAs; DE-genes, differentially expressed genes.

The GO analysis and KEGG pathway analysis were performed to investigate the biological function of the targeted genes of circRNAs. As shown in **Figures 5A, B**, the GO analysis demonstrated that the most significantly upregulated biological process was involved in the cellular process (GO:0009987), while the most significantly downregulated biological process was involved in the establishment of localization in cell (GO:0051649). In the cellular component analysis, the most significantly upregulated and downregulated item was both “intracellular” (GO:0005622). As for the molecular function analysis, the most significantly upregulated and downregulated items were “binding” (GO:0005488) and “protein binding” (GO:0005515), respectively. In KEGG pathway analysis (**Figures 5C, D**), 10 upregulated and 10 downregulated pathways with the most significant differences were identified. The upregulated pathways principally comprised the ubiquitin-mediated proteolysis, progesterone-mediated oocyte maturation, protein processing in the endoplasmic reticulum and small cell lung cancer, and the downregulated pathways principally comprised the calcium signaling pathway, glucagon signaling pathway and insulin signaling pathway.

### 3.5 Identification of circRNA-targeting miRNAs and construction of circRNA-miRNA-mRNA networks

**Figure 6:**
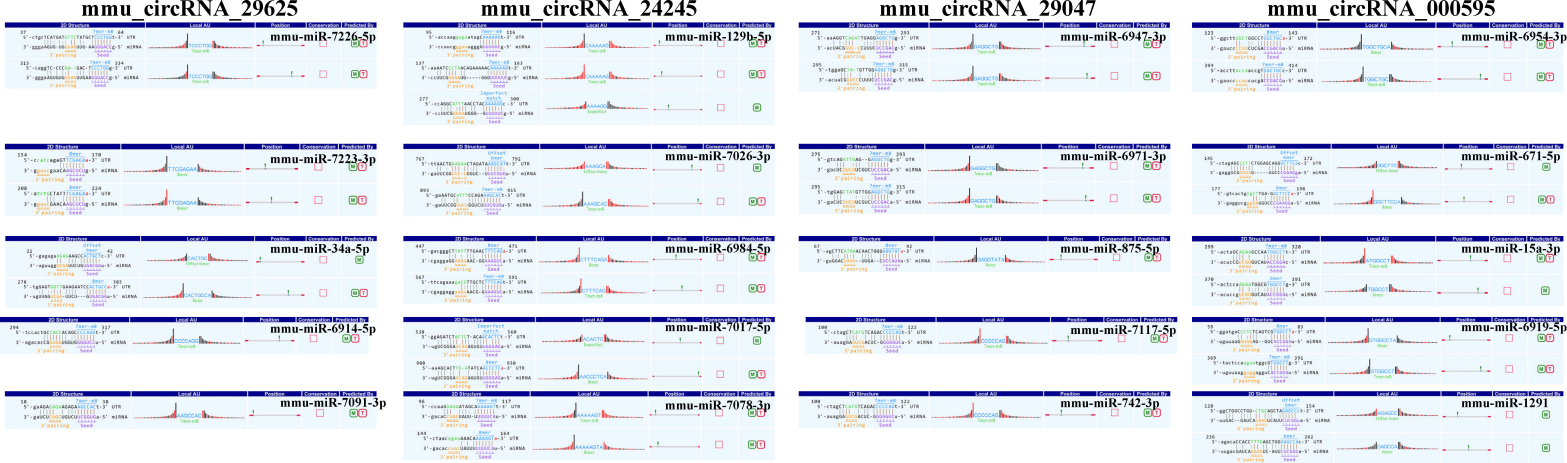
The direct interaction of the four most significantly upregulated and downregulated exonic circRNAs with their related miRNAs predicted by TargetScan and miRanda. M, miRanda; T, TargetScan.

**Figure 7:**
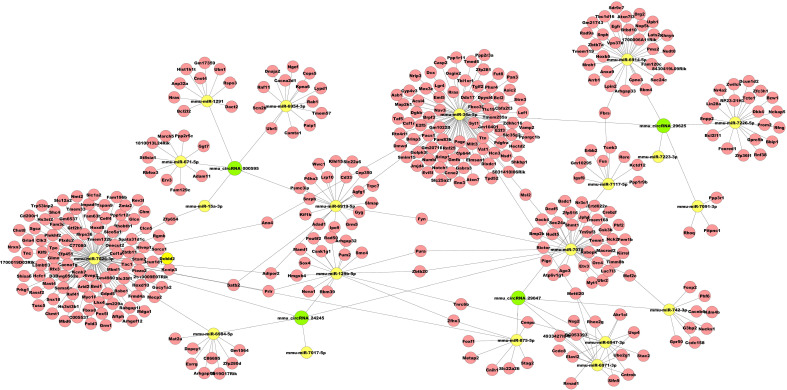
Representative circRNA-miRNA-mRNA network constructed by Cytoscape software.

To preliminarily explore whether the DE-circRNAs can function as miRNA sponges, we identified the top 5 miRNAs that may bind to it based predicted by Arraystar’s home-made miRNA target prediction software based on TargetScan and miRanda. The predicted interaction sites of the most significantly upregulated exonic circRNA-mmu_circRNA_29625 and mmu_circRNA_24245, and the most significantly downregulated exonic circRNA-mmu_circRNA_29047 and mmu_circRNA_000595 were displayed in **Figure 6**. To better explore and predict the underlying functions of DE-circRNAs during the process of HO formation, a circRNA-miRNA-mRNA regulatory network was constructed with the above circRNAs as decoys (**Figure 7**). The network suggested that circRNAs could indirectly regulate miRNA-target genes by competitively binding to miRNA.

## 4 Discussion

CircRNAs are conserved endogenous products and characterized by a covalently closed loop structure without the 5’ to 3’ polarity, which play critical functions in the biological process (16). The roles of circRNAs in skeletal disorders, including bone defects, osteoporosis, osteoarthritis and osteonecrosis of the femoral head, have been reported (13–15). However, few data are available concerning whether the expression of circRNA changes in traumatic HO, and no circRNA regulatory network has been established in traumatic HO. Systemic exploration of the circRNA profile in the traumatic HO tissues may provide a new perspective for investigating the mechanism of this pathological osteogenesis in terms of circRNAs. In the present study, we identified a total of 168 upregulated and 323 downregulated circRNAs in HO lesions through microarray assay. Among them, 10 DE-circRNAs were randomly selected for validation the results of microarray assay using qRT-PCR. The expression trends of these circRNAs were similar to the microarray analysis results. Our results first identified new circRNAs that may participate in the traumatic HO formation, providing novel treatment targets and diagnostic markers for this thorny disease.

In addition, GO enrichment and KEGG pathway analysis were also conducted to annotate the predicted target genes of circRNAs functionally. GO analysis revealed that these DE-circRNAs were involved in several cellular processes, including regulation of the cellular metabolic process, nitrogen compound metabolic process and cellular macromolecule metabolic process. In addition, KEGG enrichment analysis revealed that the upregulated pathways included the ubiquitin-mediated proteolysis, progesterone-mediated oocyte maturation, protein processing in the endoplasmic reticulum and small cell lung cancer, and downregulated pathways included the calcium signaling pathway, glucagon signaling pathway and insulin signaling pathway had a close relationship with the DE-circRNAs in HO samples. Among the detected pathways, activating the ubiquitin-mediated proteolysis has been reported to have facilitating effects on osteogenic differentiation (24). Thus, we speculate that the DE-circRNAs may be involved in the process of pathological osteogenesis during the HO formation *via* regulating different signaling pathways.

Recently, studies on the regulation of non-coding RNA in traumatic HO have increased, particularly miRNA. Geng et al. demonstrated that overexpression of mechanical sensitive miR-337-3p could alleviate ectopic ossification in a rat tendinopathy model *via* targeting IRS1 and Nox4 of tendon-derived stem cells (25). Tu et al. found that miR-203 could inhibit traumatic HO by targeting Runx2 (26). Jaira et al. identified that miR-1 and miR-206 were upregulated in samples obtained from HO-positive patients. The upregulation of miR-1 and miR-206 could enhance the differentiation of bone-forming cells (osteoblasts) and mineralization of human multipotent progenitor cells *via* targeting Sox9 (27). As circRNAs could negatively regulate the miRNAs upon target mRNAs as sponges or function with RNA binding protein (RBP) to regulate their parent genes (17), we examined circRNA-miRNA interactions by bioinformatics analysis, and discovered that each selected circRNA, containing at least one miRNA binding sites, was able to interact with several miRNAs. The results suggested that circRNAs participated in traumatic HO formation putatively through targeted miRNA and indirectly regulated gene expression.

To further understand the possible regulatory mechanism of DE-circRNAs in traumatic HO, the circRNA-miRNA-mRNA networks were established for mmu_circRNA_29625 mmu_circRNA_24245, which were the most significantly upregulated exonic circRNAs, and mmu_circRNA_29047 and mmu_circRNA_000595, which were the most significantly downregulated exonic circRNAs. The networks served as a shred of credible evidence that the exonic DE-circRNA participate in the pathogenesis of HO by negatively regulating the miRNAs upon target mRNAs as sponges. Among the predicted miRNAs, the mmu_miR-34a-5p, which could interact with mmu_circRNA_29625, has been reported to inhibit osteogenic differentiation and *in vivo* bone formation of osteoblasts by mediating the reduction of JAG1 expression (28). Therefore, mmu_circRNA_29625 has the potential to stimulate the osteogenic differentiation of TMPCs through competitive inhibition of mmu_miR-34a-5p, and then promote the HO formation. Furthermore, the potential target genes of mmu_circRNA_29625-mmu_miR-34a-5p were also shown in the network, such as Notch1, which are critical members of the Notch pathway served as the single-pass transmembrane receptor (29). The activation of Notch1 has been reported to accelerate the differentiation of osteoblasts to osteocytes (30, 31) and participate in craniofacial, axial, and appendicular skeletal development and fracture healing (32, 33). To our knowledge, few data were available concerning the roles of mmu_circRNA_29625, mmu_miR-34a-5p and Notch pathway in HO, and further biological experiments are warranted to investigate their possible roles.

In addition, mmu_circRNA_24245 could also regulate mmu_miR-129b-5p binding to the Sox4 gene exhibited in the network. Previous research has demonstrated that inhibiting miR-129-5p expression could activate the BMP2/Smad pathway, thereby showing stimulative effects on osteogenic differentiation of human periodontal ligament stem cells, similar to TMPCS which contribute to HO under inflammatory insult (34). Sox4 is expressed in numerous progenitor cell types, including skeletal progenitors, and is necessary for cell survival during mesenchyme formation (35). Besides, Sox4 may promote cell migration and TGF-β-induced epithelial-to-mesenchymal transition (EMT) (36), which has been proven to participate in HO formation and occurs in response to inflammatory cytokines, such as BMP-4 and TGF-β (37, 38). Based on the above crosstalk, we thus speculate that the mmu_circRNA_24245-mmu_miR-129b-5p-Sox4 axis may also be a critical signaling that promotes traumatic HO formation.

Some potential limitations also existed in our study. Firstly, we have only provided a new perspective for investigating the mechanism of HO formation in terms of circRNAs, and it is necessary to explore the potential functions of these DE-circRNAs by further research. Secondly, because male patients are more likely to develop HO than females (39), we only used male mice to establish the HO model to avoid the effect of gender difference on circRNA expression. It is necessary to further explore the differences in circRNA expression between different genders. Finally, the HO samples were only collected at one time point after surgery. Further research screening out the DE-circRNAs based on a time axis after surgery might provide more clinical significance for diagnosing or monitoring traumatic HO formation.

## Conclusion

In conclusion, our study first demonstrated the expression profiles and predicted the potential functions of circRNAs in mice traumatic HO. The results provide new clues for the elucidation of mechanisms as well as novel potential peripheral biological diagnostic markers and therapeutic targets for traumatic HO.

## Data availability statement

The datasets presented in this study can be found in Gene Expression Omnibus (GEO) with accession number GSE217301.

## Ethics statement

All experiments were approved by the Experimental Animal Welfare Ethics Committee of Zhongnan Hospital of Wuhan University under animal protocol number ZN2021153.

## Author contributions

QL, ZL and AY participated in the study design and conception. ZW, XY and YL implemented the experiment and collected and analyzed the data. ZW and XY interpreted the data and drafted the manuscript. All authors contributed to the article and approved the submitted version.
